# Supplementary material for: Exploring the potential of dental calculus to shed light on past human migrations in Oceania
Source: Nat Commun. 2024 Nov 24;15:10191. doi: 10.1038/s41467-024-53920-z (PMC11586442; doi:10.1038/s41467-024-53920-z)
Supplement: Supplementary file 1 — Supplementary Information [file 41467_2024_53920_MOESM1_ESM.pdf]

Supplementary material for

Exploring the potential of dental calculus to shed light on past human migrations in Oceania

Irina M. Velsko, Zandra Fagernäs, Monica Tromp, Stuart Bedford, Hallie R. Buckley, Geoffrey Clark, John Dudgeon, James Flexner, Jean-Christophe Galipaud, Rebecca Kinaston, Cecil M. Lewis, Jr, Elizabeth Matisoo-Smith, Kathrin Nägele, Andrew T. Ozga, Cosimo Posth, Adam B. Rohrlach, Richard Shing, Truman Simanjuntak, Matthew Spriggs, Anatauarii Tamarii, Frédérique Valentin, Edson Willie, Christina Warinner*

Supplementary Figures

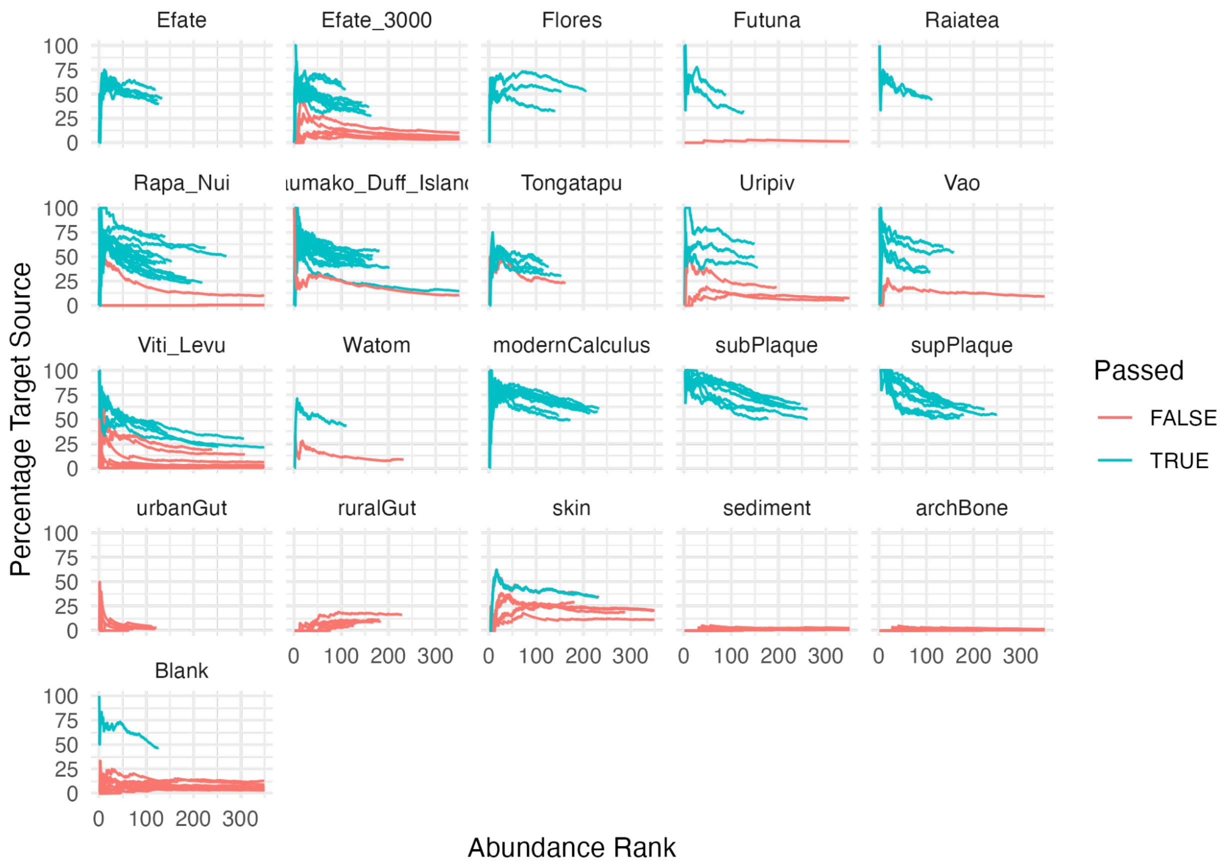

Supplementary Figure 1. Cumulative percent decay (cuperdec) curves for newly sequenced samples from the Pacific presented in this study. Samples are grouped by island. Passed True indicates a sample passed the cut-off and is well-preserved, while Passed False indicates a sample did not pass the cut-off and is not well-preserved.

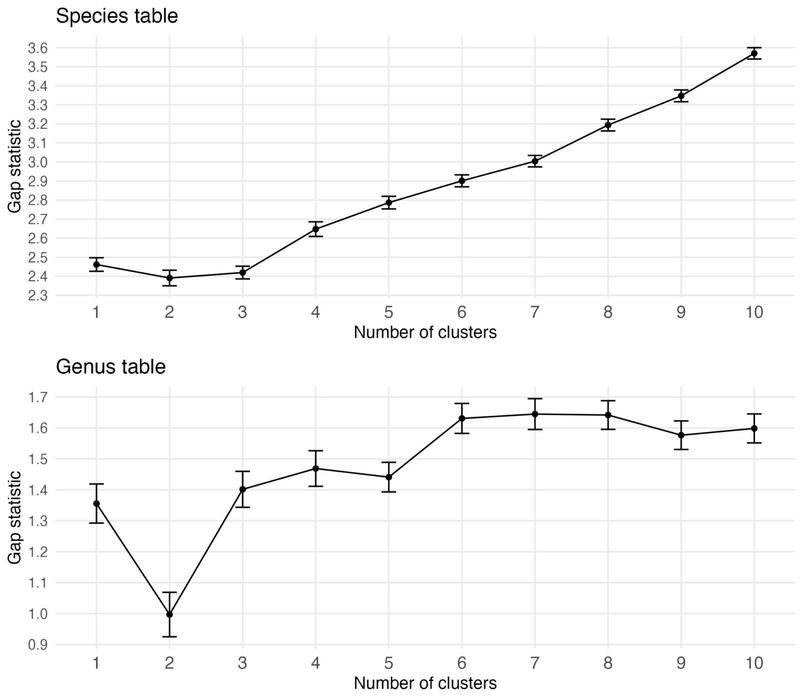

Supplementary Figure 2. Gap statistic to test for the optimal number of sample clusters in the Pacific calculus dataset. The upper panel is based on the species table, while the lower panel is based on the genus table. The Gap statistic tests the goodness of fit of the number of clusters in a dataset. The optimal number of clusters is determined as the cluster before the first drop in value of the Gap statistic. When using either the species table or the genus table, the first drop in the Gap statistic occurs at 2 clusters, such that the optimal number of clusters in the samples is 1. Error bars indicate standard error of the mean based on 500 bootstrap replicates.

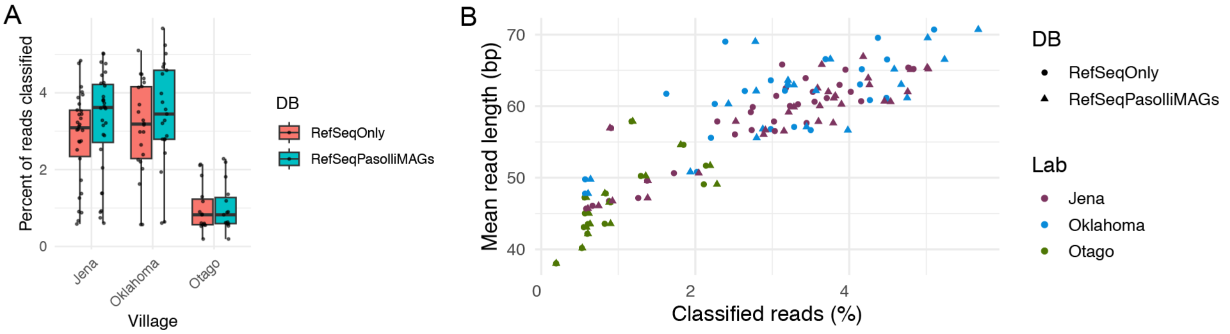

Supplementary Figure 3. Inability to increase read taxonomic assignment by using a database with novel metagenome-assembled genomes (MAGs) not found in the NCBI RefSeq database. (**A**) Percent of reads per sample that were assigned taxonomy when using the taxonomic profiler Kraken2 and a database consisting of RefSeq genomes only, or a database of the same RefSeq genomes plus MAGs published by Pasolli, et al. (2019). (**B**) Correlation between the percentage of classified reads per sample and the average read length in each sample. Samples with longer average read length have higher average percent of reads assigned taxonomy.

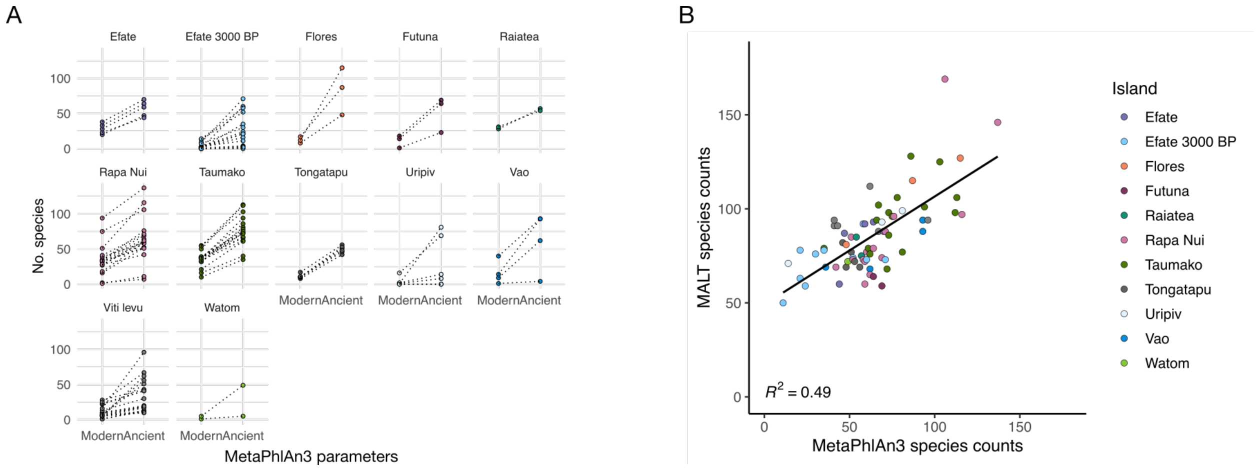

Supplementary Figure 4. Species counts by MetaPhlAn3. (**A**) Number of species detected per sample by MetaPhlAn3 using modern or ancient parameters for the bowtie2 mapping step. (**B**) Concordance between the number of species detected by MALT after filtering and by MetaPhlAn3 run with ancient parameters.

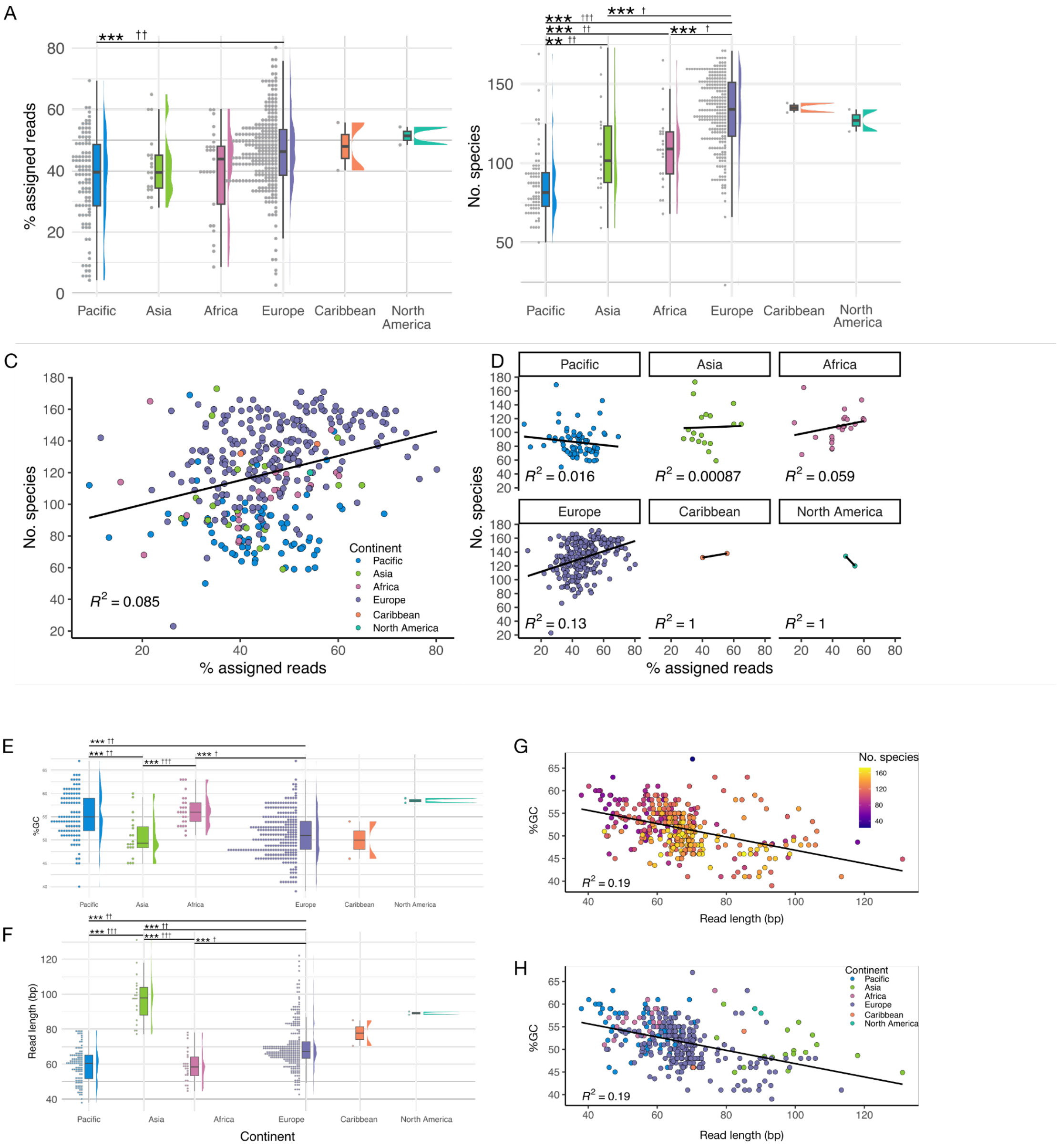

Supplementary Figure 5. Read and species assignment rates. (**A**) The percentage of reads in each sample that was assigned taxonomy by MALT, grouped by continent of origin. (**B**) The number of species detected in each sample after filtering. (**C**) Number of species and percent of assigned reads in samples. (**D**) Same as **C** but separated by continent. The Pacific samples have a slight negative association between the percent of assigned reads and the number of detected species. (**E**) Average GC content of the reads per sample, grouped by continent. (**F**) Average read length per sample, grouped by continent. (**G**) Average GC content, read length per sample, and number of species assigned per sample, are weakly negatively correlated. (**H**) Same as (**G**) but colored by continent of origin. ** $p < 0.01$, *** $p < 0.001$, † effect size < 0.3, †† effect size >= 0.3, <= 0.5, ††† effect size > 0.5.

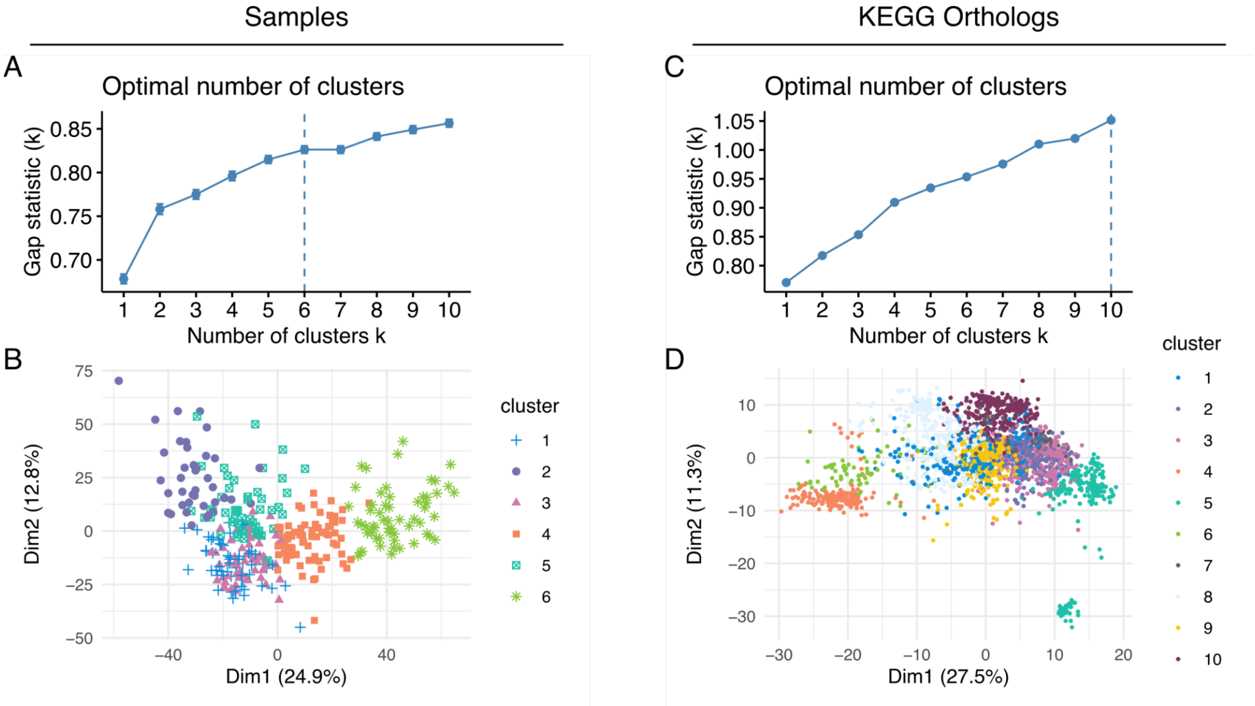

Supplementary Figure 6. Gap statistic to test for the optimal number of sample clusters (**A**, **B**) and KEGG Ortholog clusters (**C**, **D**) in the KEGG Ortholog dataset. The Gap statistic tests the goodness of fit of the number of clusters in a dataset. The optimal number of clusters is determined as the cluster before the first drop in value of the Gap statistic. (**A**) Gap statistic indicates that 6 clusters is optimal for samples. (**B**) Sample cluster plot. Samples are colored and shaped by the cluster determined by the Gap statistic. As clusters are not clearly defined, but the samples appear to take 2 trajectories in the plot (toward the upper left corner and toward the upper right corner), a sample cluster number of 2 was selected for hierarchical clustering. (**C**) Gap statistic indicates that 10 clusters are insufficient to cluster KEGG orthologs. (**D**) KEGG ortholog cluster plot. KEGG orthologs are colored by the cluster determined by the Gap statistic. As clusters are not clearly delineated, but there appear to be 3 clusters by visual inspection (a large central cluster, a small cluster in the bottom right corner (half of cluster 5), and a not-very-distinctly separated cluster on the left side (clusters 4 and 6), a KEGG ortholog cluster number of 3 was selected for hierarchical clustering.

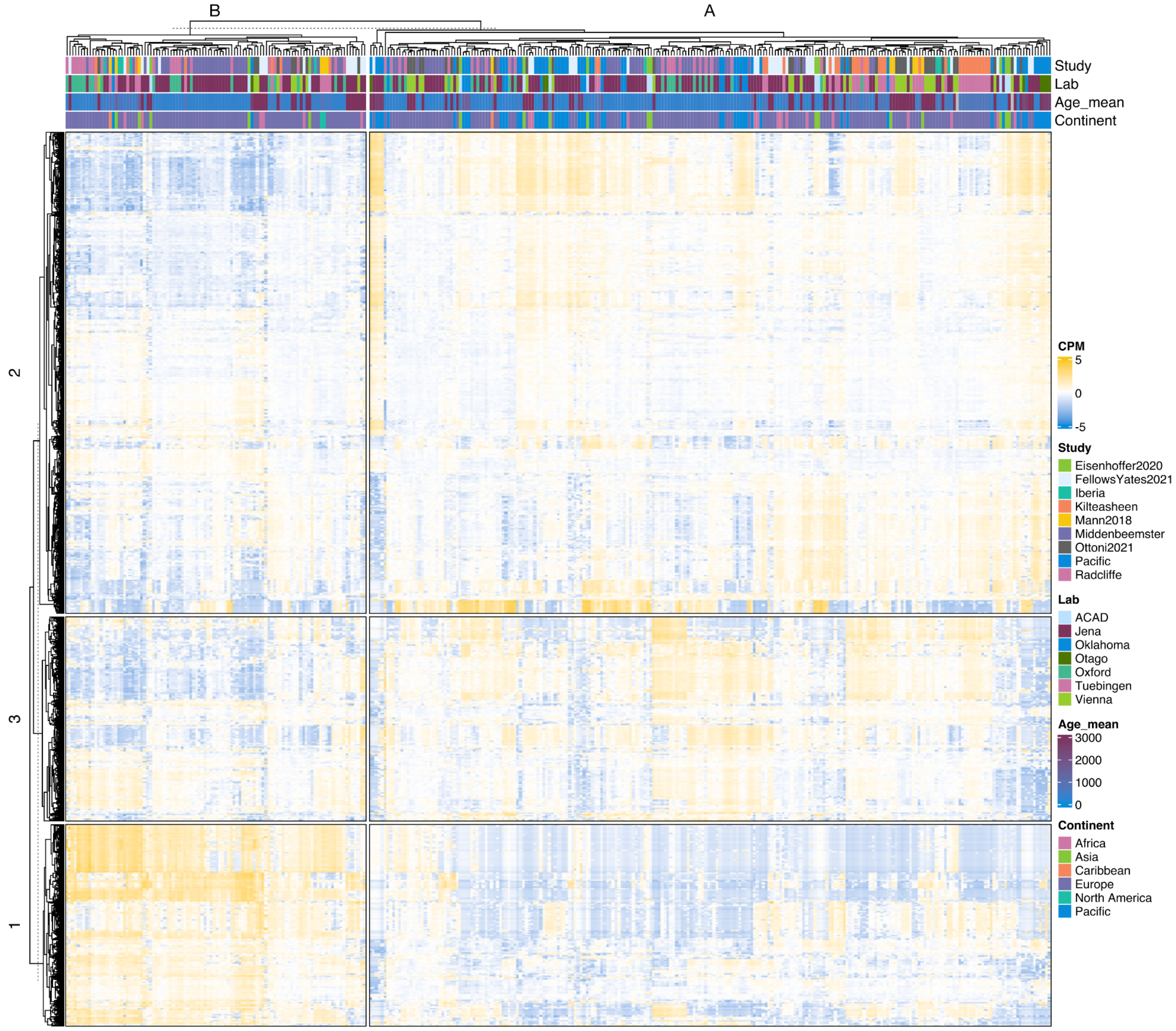

Supplementary Figure 7. Hierarchically clustered heatmap of KEGG ortholog (y-axis) abundance (CLR-transformed copies per million) in all samples (x-axis), same as main Figure 4A, but with additional sample metadata shown across the top including the lab in which sample processing was performed, and the study in which samples were originally published.

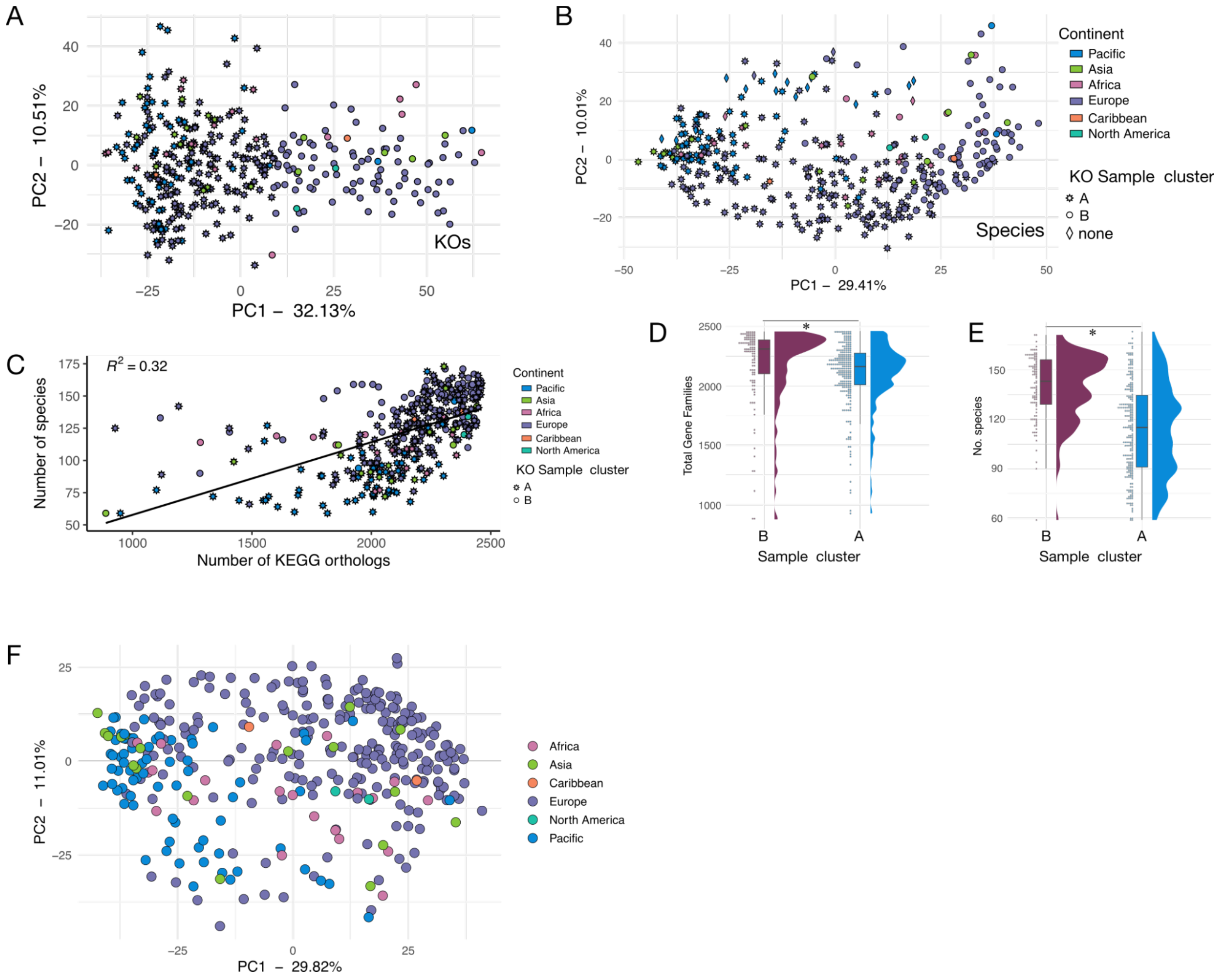

Supplementary Figure 8. Association between KEGG ortholog counts and species counts in samples. (**A**) PCA based on KEGG ortholog abundance, colored by continent and shaped by cluster from hierarchical clustering shown in main Figure 4. (**B**) PCA based on species abundance (same as main Figure 3A) colored by continent and shaped by cluster from KEGG ortholog clustering, same as panel A. The diamond samples with no cluster were excluded from KEGG Ortholog analyses. (**C**) Correlation between the number of species and the number of KEGG orthologs detected in a sample. (**D**) The number of KEGG orthologs in each sample, grouped by the sample cluster from hierarchical clustering. (**E**) The number of species, from taxonomic profiling, in each sample, grouped by the sample cluster from hierarchical clustering. * $p < 0.05$. (**F**) PCA based on the species table used to generate main text Figure 3A, from which *Ottowia* species were filtered out.

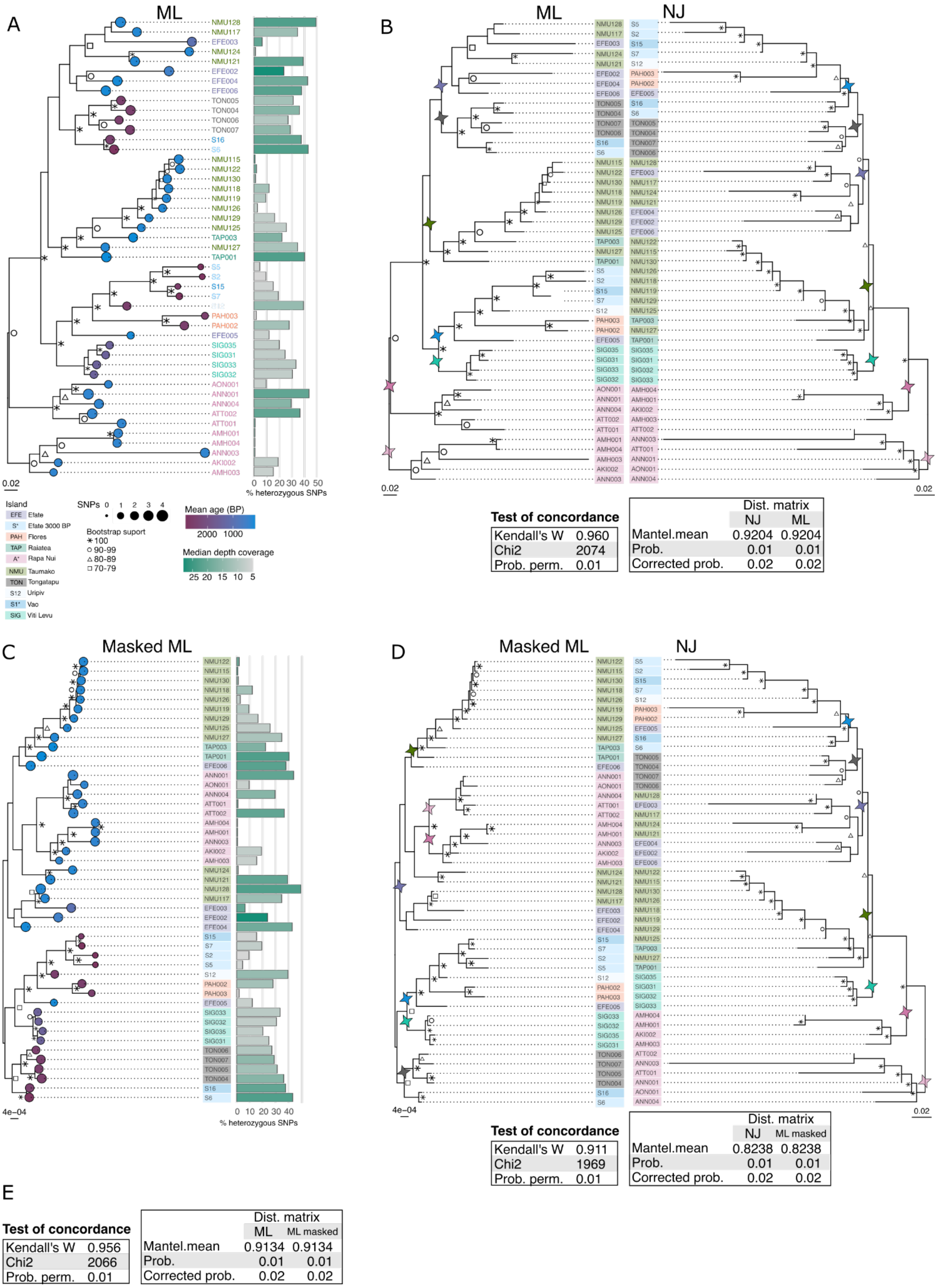

Test of concordance (B)

Kendall's W	0.960
Chi2	2074
Prob. perm.	0.01

	Dist. matrix	
	NJ	ML
Mantel.mean	0.9204	0.9204
Prob.	0.01	0.01
Corrected prob.	0.02	0.02

Test of concordance (D)

Kendall's W	0.911
Chi2	1969
Prob. perm.	0.01

	Dist. matrix	
	NJ	ML masked
Mantel.mean	0.8238	0.8238
Prob.	0.01	0.01
Corrected prob.	0.02	0.02

Test of concordance (E)

Kendall's W	0.956
Chi2	2066
Prob. perm.	0.01

	Dist. matrix	
	ML	ML masked
Mantel.mean	0.9134	0.9134
Prob.	0.01	0.01
Corrected prob.	0.02	0.02

Supplementary Figure 9. Phylogenetic trees for *Anaerolineaceae* bacterium oral taxon 439 built with maximum likelihood (ML) methods show high similarity to those built the neighbor-joining (NJ) method. (**A**) ML tree of *Anaerolineaceae* bacterium oral taxon 439 built using the same SNP alignment as for main text figure 5B. (**B**) Comparison of tree branching patterns between the ML tree of panel A and the NJ tree of main text figure 5A. Colored stars indicate branches that include the same, or nearly all of the same samples. The table below contains values for the test of correspondence (Kendall's W) between the two trees. (**C**) ML tree of *Anaerolineaceae* bacterium oral taxon 439 built using the masked alignment produced by Gubbins. (**D**) Comparison of tree branching patterns between the masked alignment-based tree of panel C and the NJ tree of main text figure 5A. Colored stars indicate branches that include the same, or nearly all of the same samples. The table below contains values for the test of correspondence (Kendall's W) between the two trees. (**E**) Test of correspondence between the SNP alignment ML tree and the masked SNP alignment ML tree.

A

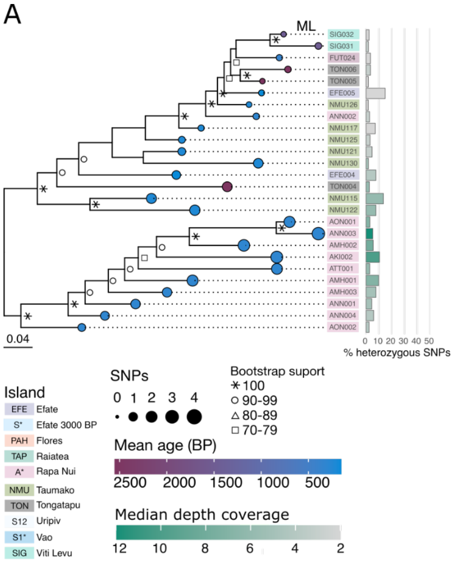

B

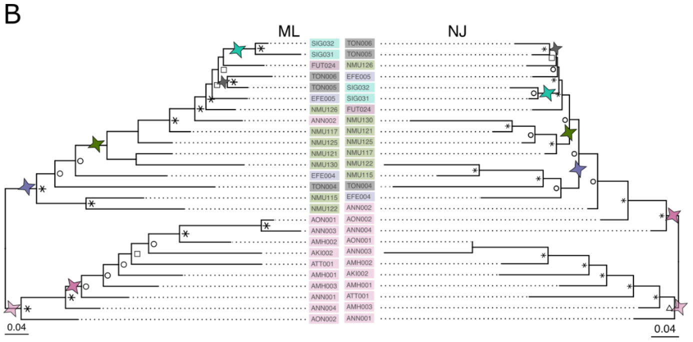

Test of concordance

Kendall's W	0.909
Chi2	589
Prob. perm.	0.01

	Dist. matrix	
	NJ	ML
Mantel.mean	0.8180	0.8180
Prob.	0.01	0.01
Corrected prob.	0.02	0.02

C

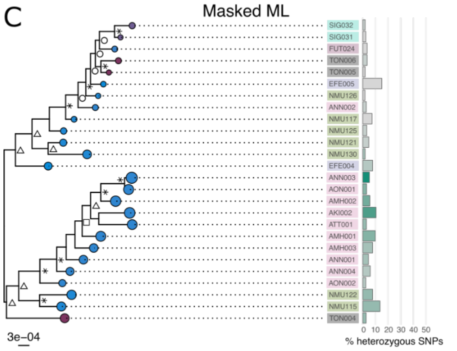

D

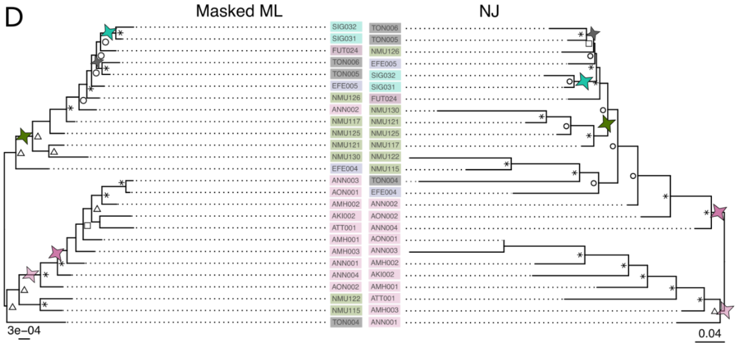

Test of concordance

Kendall's W	0.8395
Chi2	544
Prob. perm.	0.01

	Dist. matrix	
	NJ	ML masked
Mantel.mean	0.6790	0.6790
Prob.	0.01	0.01
Corrected prob.	0.02	0.02

E

Test of concordance

Kendall's W	0.9839
Chi2	637
Prob. perm.	0.01

	Dist. matrix	
	ML	ML masked
Mantel.mean	0.9678	0.9678
Prob.	0.01	0.01
Corrected prob.	0.02	0.02

Supplementary Figure 10. Phylogenetic trees for *Tannerella forsythia* built with maximum likelihood (ML) methods show high similarity to those built the neighbor-joining (NJ) method. (**A**) ML tree of *T. forsythia* built using the same SNP alignment as for main text figure 5B. (**B**) Comparison of tree branching patterns between the ML tree of panel A and the NJ tree of main text figure 5B. Colored stars indicate branches that include the same, or nearly all of the same samples. The table below contains values for the test of correspondence (Kendall's W) between the two trees. (**C**) ML tree of *T. forsythia* built using the masked alignment produced by Gubbins. (**D**) Comparison of tree branching patterns between the masked alignment-based tree of panel C and the NJ tree of main text figure 5B. Colored stars indicate branches that include the same, or nearly all of the same samples. The table below contains values for the test of correspondence (Kendall's W) between the two trees. (**E**) Test of correspondence between the SNP alignment ML tree and the masked SNP alignment ML tree.

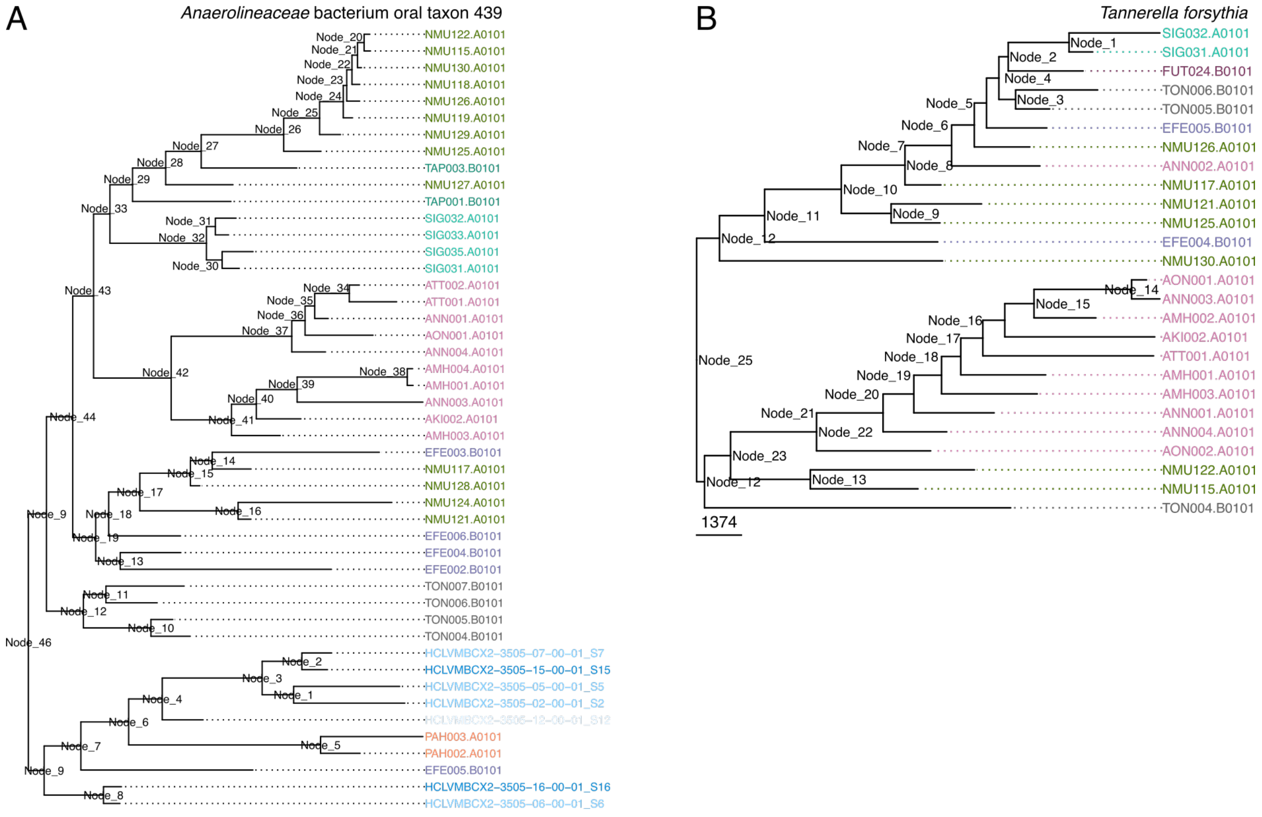

Supplementary Figure 11. SNP-based alignment trees produced by Gubbins. (**A**) *Anaerolineaceae* bacterium oral taxon 439. (**B**) *Tannerella forsythia*. Node labels correspond to those in Supplementary tables S6 and S7 for panels A and B, respectively.

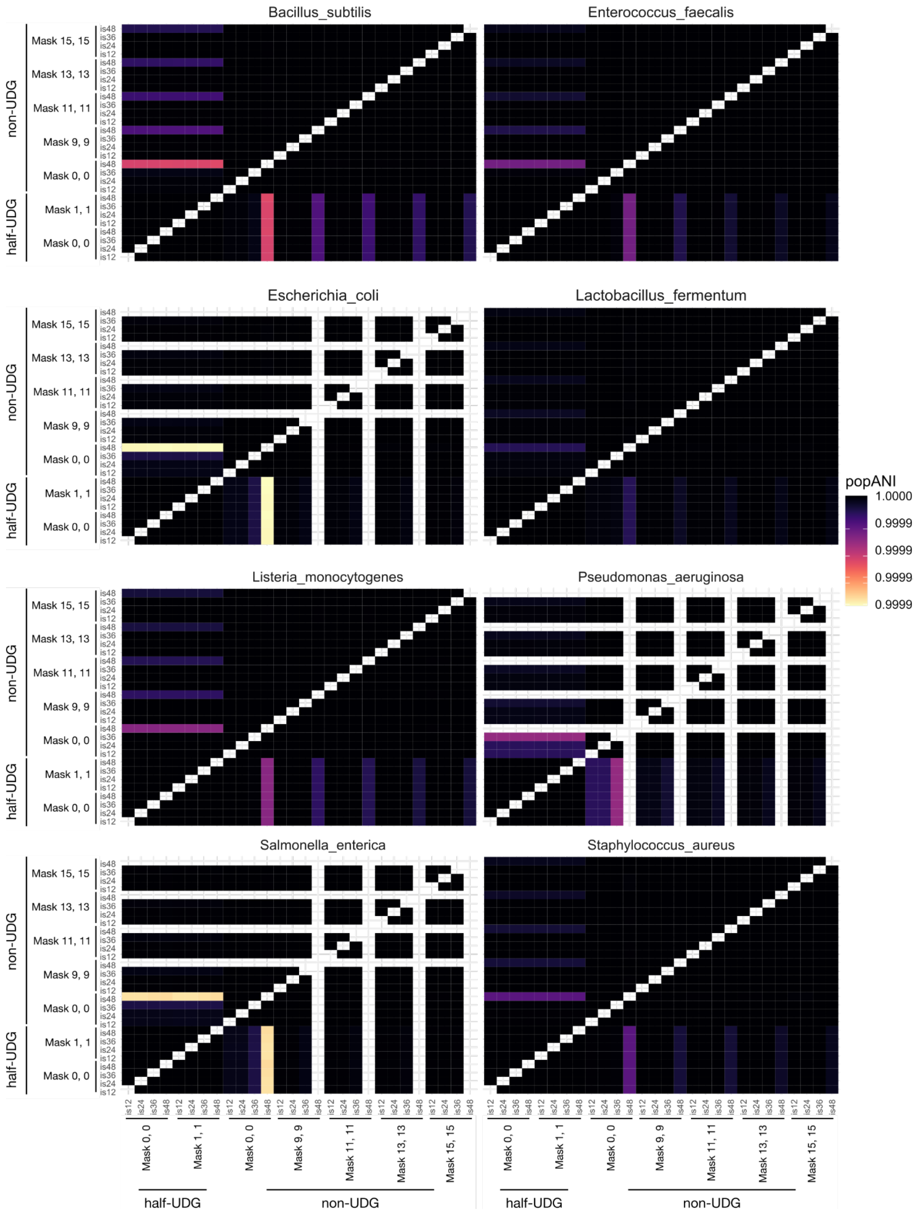

Supplementary Figure 12. Simulated metagenome parameter testing for inStrain assessment of strain diversity in ancient metagenomes. PopANI of species in a synthetic community with differing levels of aDNA damage (C-T transitions). Synthetic communities were simulated with high C-T transition rates (non-UDG) or low C-T transition rates (UDG-half). Following mapping against reference genomes, the ends of reads were masked in the mapped read bam file at different lengths along the read to see whether and how much C-T transitions affect popANI estimations. In the sample name, the numbers following “mask” indicate the number of bases from the first and last base on each read were masked, i.e. “non_udg_mask_15_15” indicates the read was simulated with high C-T transition rate and the first and last 15 bases of each mapped read were masked to hide potential damaged sites.

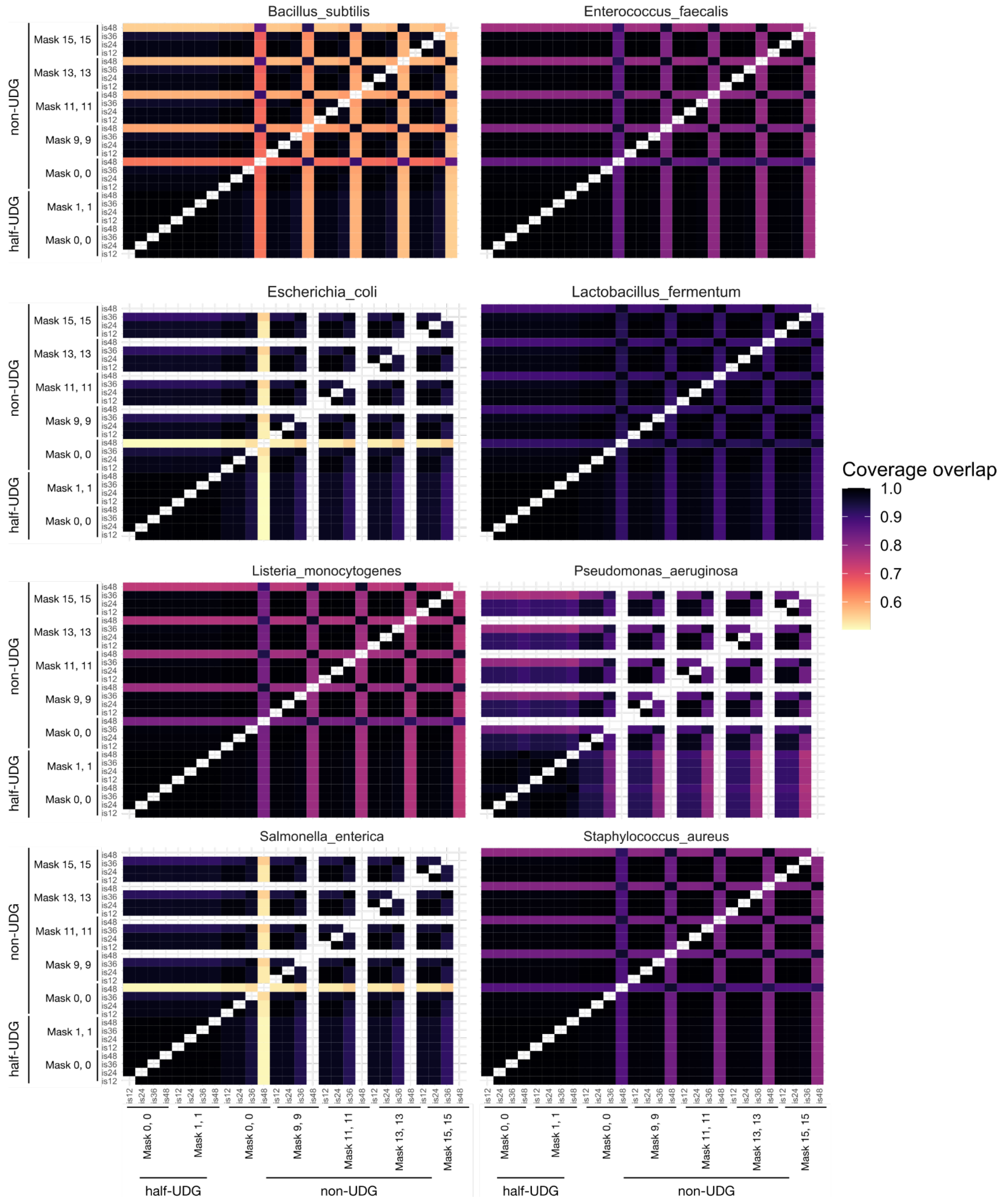

Supplementary Figure 13. Simulated metagenome parameter testing for inStrain assessment of strain diversity in ancient metagenomes. Percent of genome compared between samples in a synthetic community with differing levels of C-T transitions. Synthetic communities were simulated with high C-T transition rates (non-UDG) or low C-T transition rates (UDG-half). Following mapping against reference genomes, the ends of reads were masked in the mapped read bam file at different lengths along the read to see whether and how much C-T transitions affect popANI estimations. In the sample name, the numbers following “mask” indicate the number of bases from the first and last base on each read were masked, i.e. “non_udg_mask_15_15” indicates the read was simulated with high C-T transition rate and the first and last 15 bases of each mapped read were masked to hide potential damaged sites.

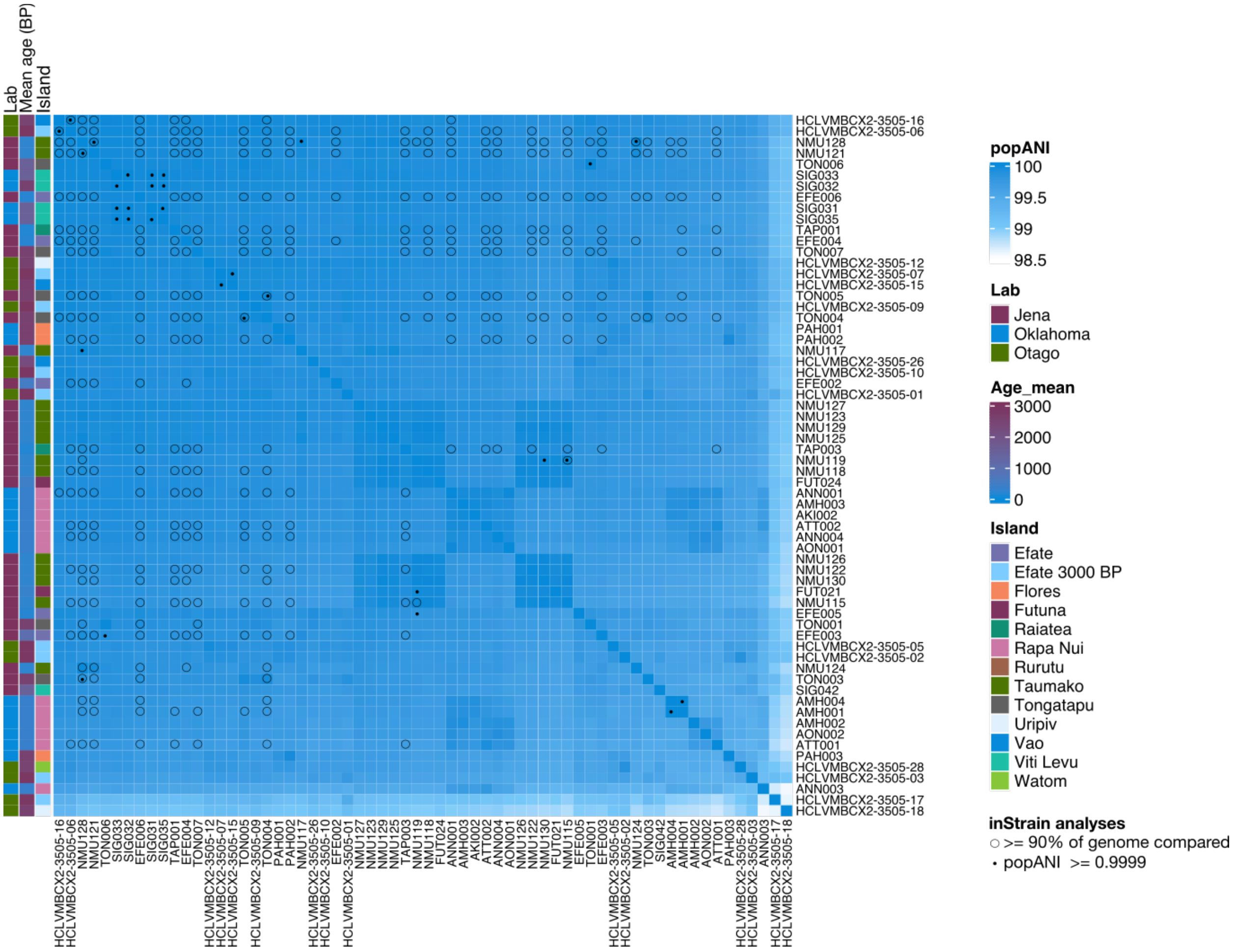

Supplementary Figure 14. Heat map showing the inStrain popANI for *Anaerolineaceae* bacterium oral taxon 439 between Pacific samples. Black dots indicate samples that have a popANI ≥ 0.9999 (range: 0.9999083 - 0.9999970), and open black circles indicate samples where ≥ 90% of the genome could be compared. Only samples AMH001 and AMH004 have a popANI > 0.99999.

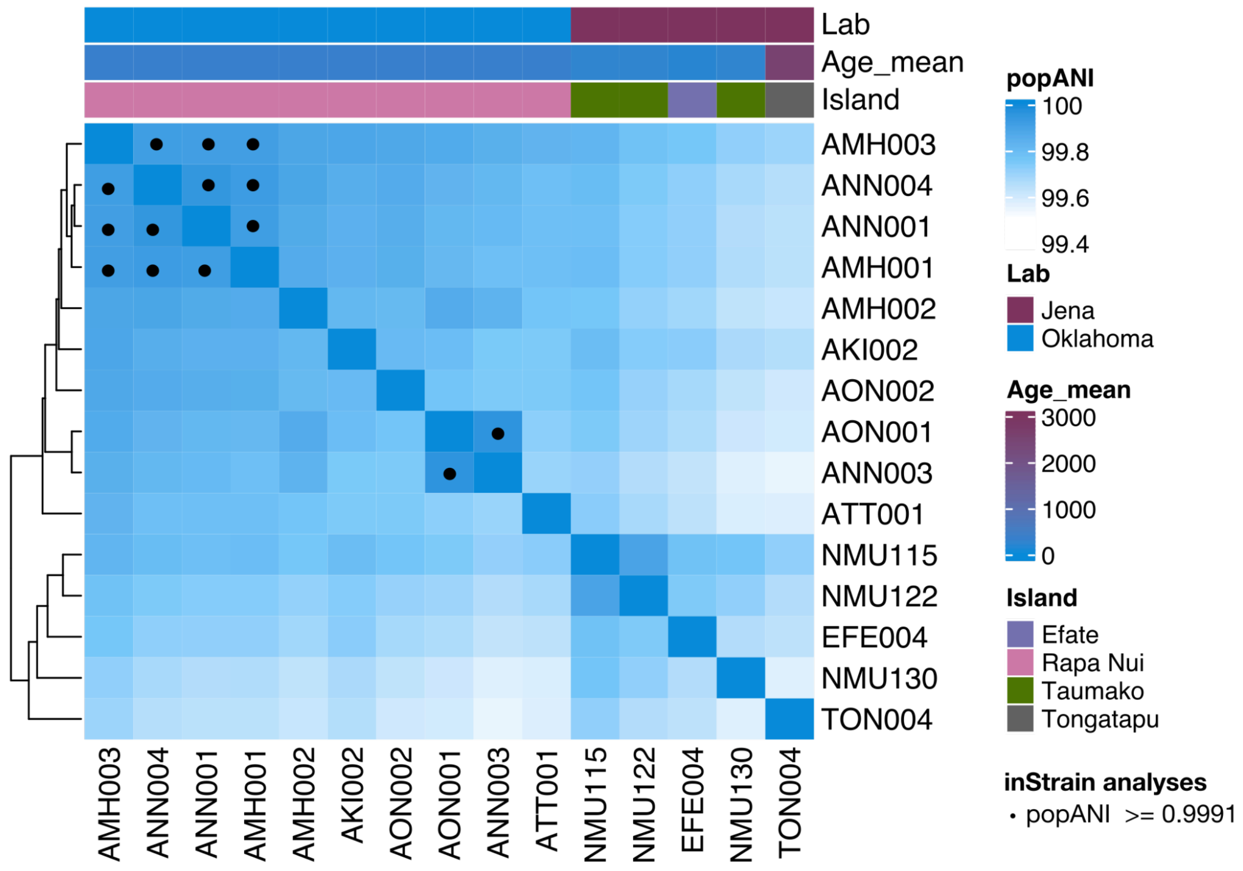

Supplementary Figure 15. Heat map showing the inStrain popANI for *Tannerella forsythia* between Pacific samples. Samples are hierarchically clustered. The percent of genome compared between each sample was at most 82%, suggesting that low coverage of this species reduces the ability to distinguish strains. Black dots indicate a popANI of > 0.9991 (range 0.9991 - 0.99955).

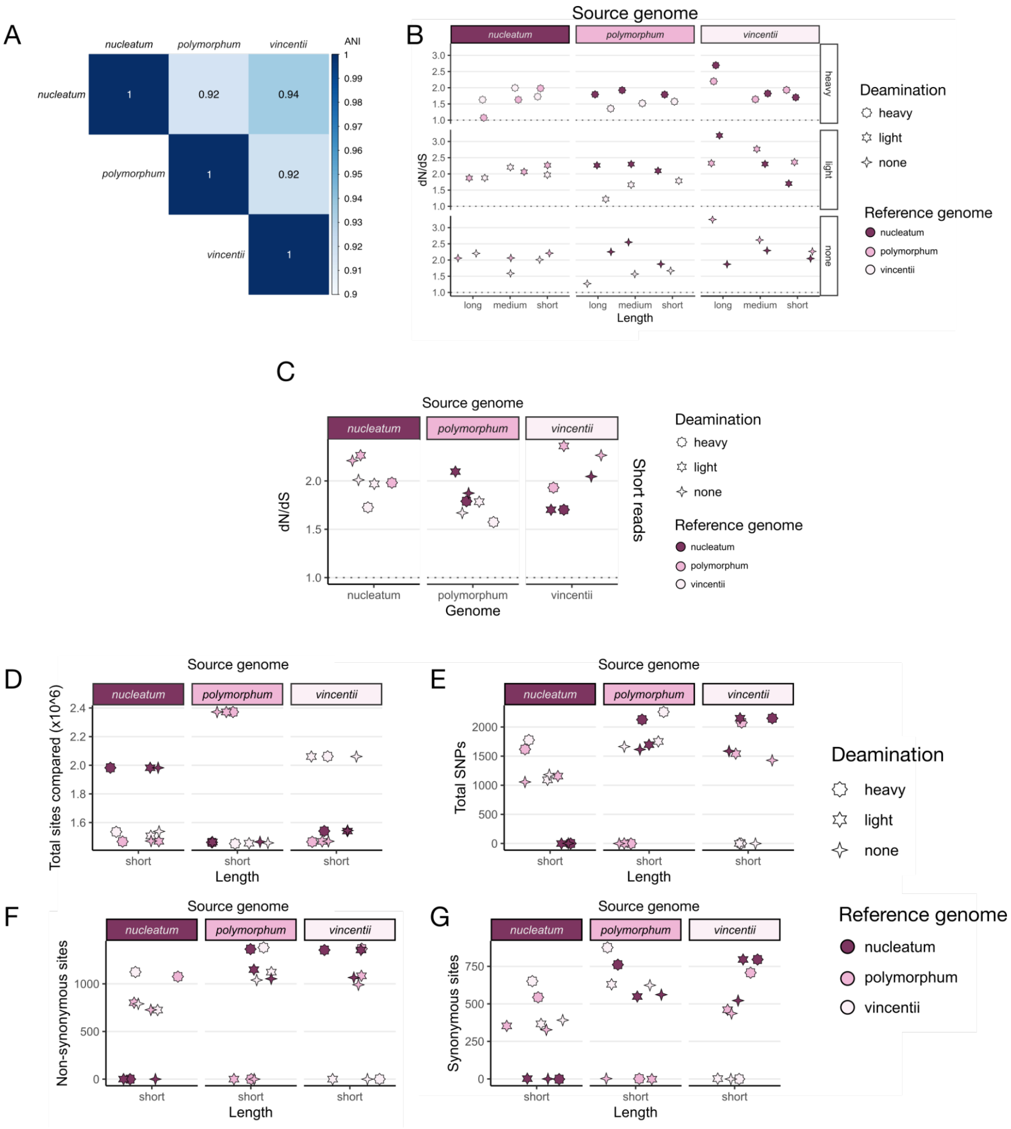

Supplementary Figure 16. *Fusobacterium nucleatum* subspecies as a test-case for dN/dS values for mapping against an incorrect but closely-related species. Three subspecies of *Fusobacterium nucleatum* (*nucleatum*, *polymorphum*, and *vincentii*) were reduced to short read sequencing data of three lengths, long, medium, or short (150bp, 75bp, or 30bp, respectively), and ancient DNA damage C-T transitions were added in a high or low amount, or left off (none, essentially modern DNA). (**A**) ANI values of each subspecieis genome compared to the other two. (**B**) dN/dS values calculated with polymut after mapping the short read data against each of the three subspecies genomes. (**C**) dN/dS of short read data of each genome mapped against the other genomes. (**D**) Total sites compared for each genome compare to the other genomes. (**E**) Total SNP's identified by polymut with default parameters. (**F**) Total non-synonymous SNPs for each genome mapped against the other genomes. (**G**) Total synonymous SNPs for each genome mapped against the other genomes.

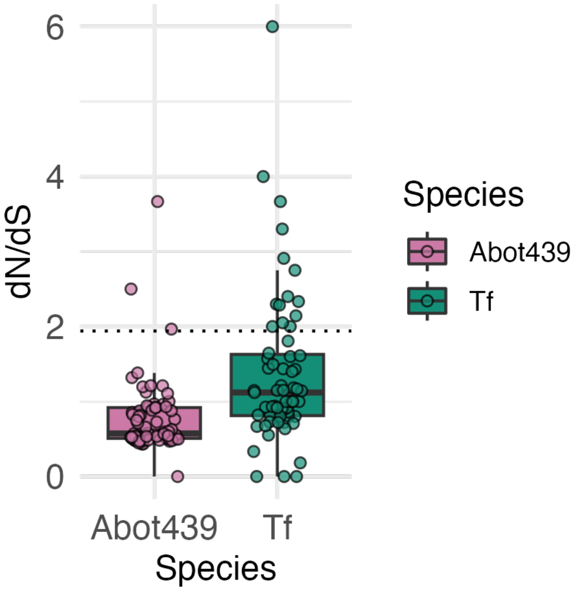

Supplementary Figure 17. The ratio of non-synonymous SNPs to synonymous SNPs (dN/dS) for all samples mapped against the *Anaerolineaceae* bacterium oral taxon 439 or *Tannerella forsythia* genome. The dotted line at 1.94 indicates the dN/dS of mapping a species to a closely-related reference genome (see Supplementary Figure 16). Abot439 - *Anaerolineaceae* bacterium oral taxon 439; Tf - *Tannerella forsythia*.

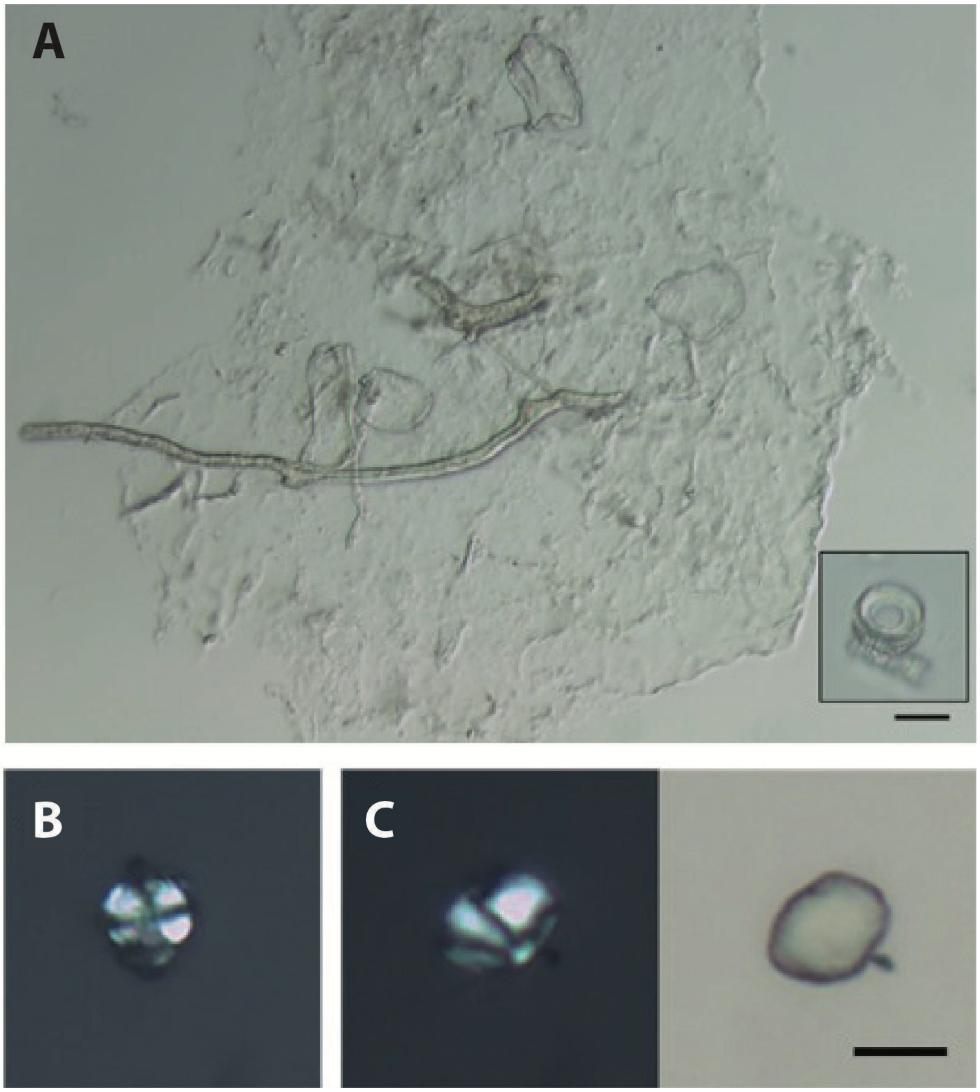

Supplementary Figure 18. Microfossils observed in dental calculus from Efate. (**A**) Fungal spores and hyphae, with inset showing two diatoms; 10 μm scale bar applies to both images. Starch granules from (**B**) EFE003.B (cross-polarized light) and (**C**) EFE006.B (left in cross-polarized light, right in transmitted light); scale bar is 10 μm and applies to both images.

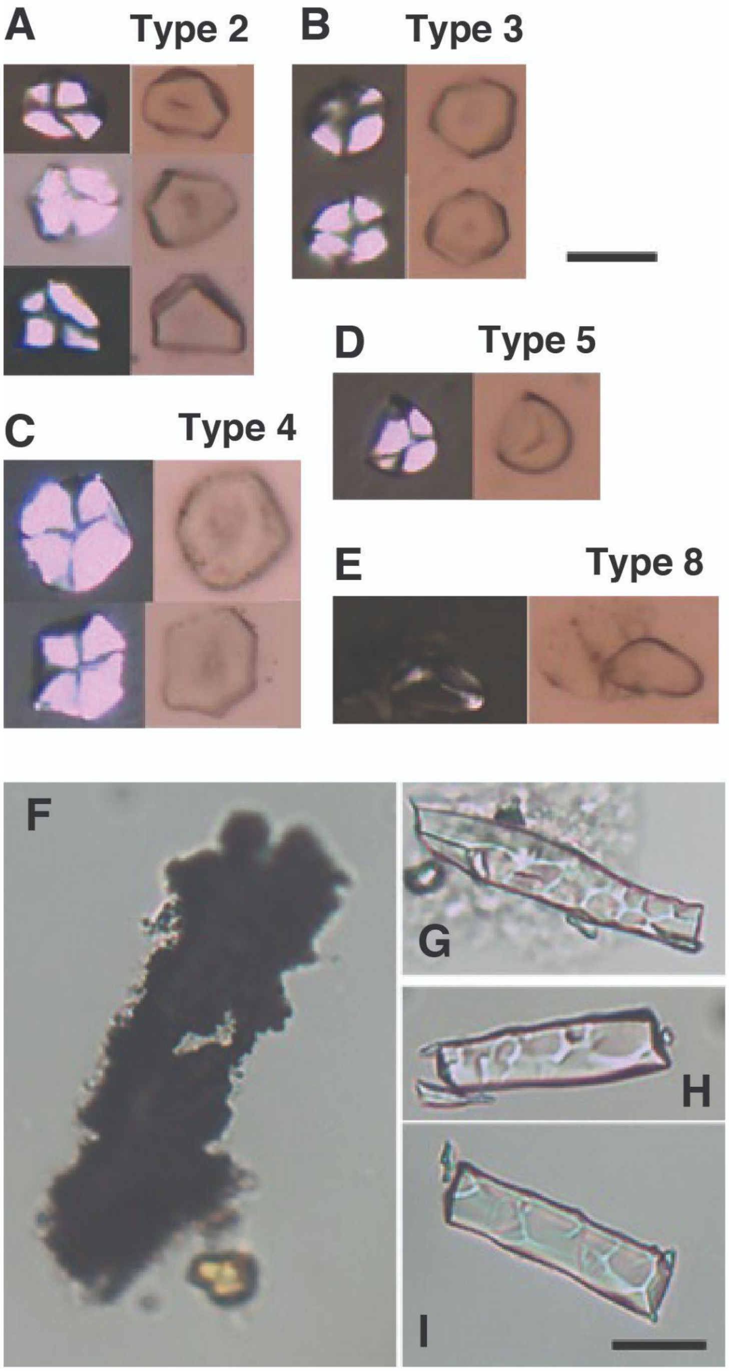

Supplementary Figure 19. Microfossils observed in dental calculus from Futuna. (**A-E**) Starch granules from sample FUT018.B (left in cross-polarized light, right in transmitted light); scale bar is 10 μm and applies to all images. (**F**) Microcharcoal and (**G-I**) phytoliths from sample FUT021.B; scale bar is 20 μm and apply to all images.

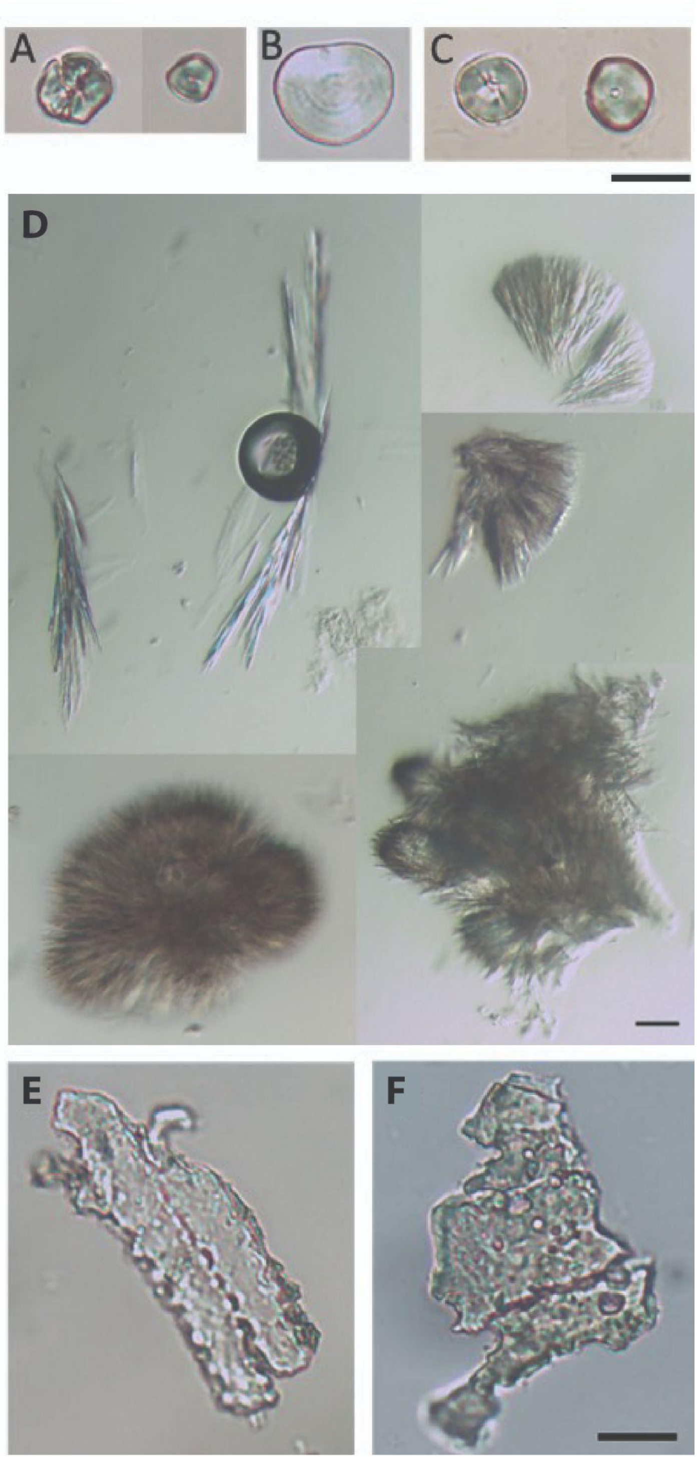

Supplementary Figure 20. Microfossils observed in dental calculus from Taumako. Starch granules from (**A**) NMU119.A, (**B**) NMU122.A and (**C**) NMU127.A; scale bar is 20 µm and applies to all samples. (**D**) Unknown microparticles from sample NMU116.A; scale bar is 20 µm and applies to all images. (**E**) Dentate elongate phytoliths from NMU122.A, and (**F**) damaged phytoliths from NMU123.A; scale bar is 20 µm and applies to both images.

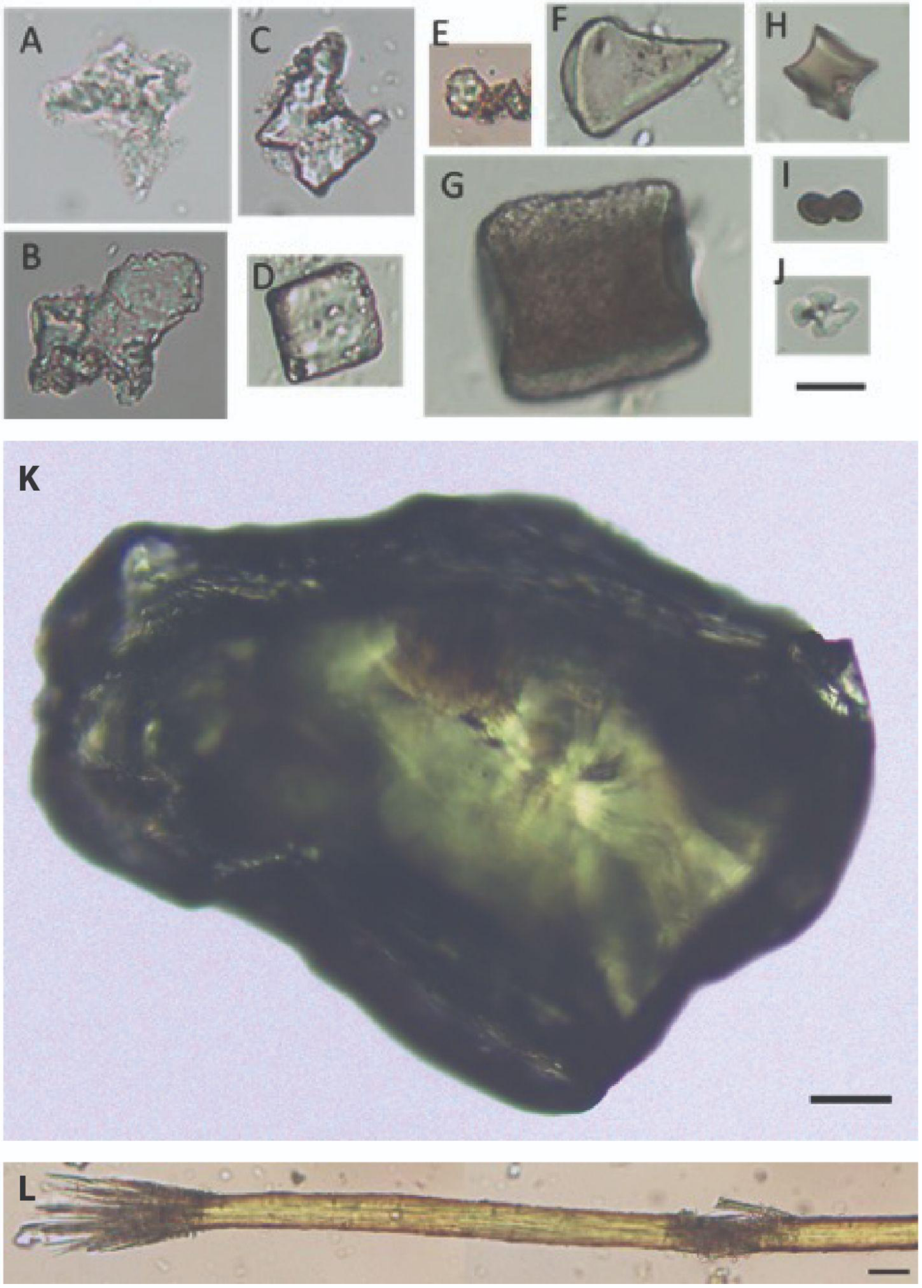

Supplementary Figure 21. Microfossils observed in dental calculus from Fiji. Phytoliths from SIG040.A (**A, B**), SIG042.A (**C**), SIG031.A (**D**), SIG045.A (**E**), SIG032.A (**F, G**), SIG036.A (**H, I, J**). **A** and **C** look similar to double peaked glume phytoliths; (**B**) is an amoeboid echinate phytolith of unknown origin; (**D**) and (**G**) are blocky type phytoliths; (**E**) is a spheroid echinate (palm) phytolith; (**F**) is an acute bulbus phytolith; (**H**) is a burnt rondel phytolith; (**I**) is a burnt bilobate phytolith; (**J**) is a cross phytolith; scale bar is 10 µm and applies to all images. (**K**) Example of the abundant olive green mineral particles found in the Fiji samples; scale bar is 50 µm. (**L**) Fiber found in sample SIG044.A; scale bar is 50 µm.

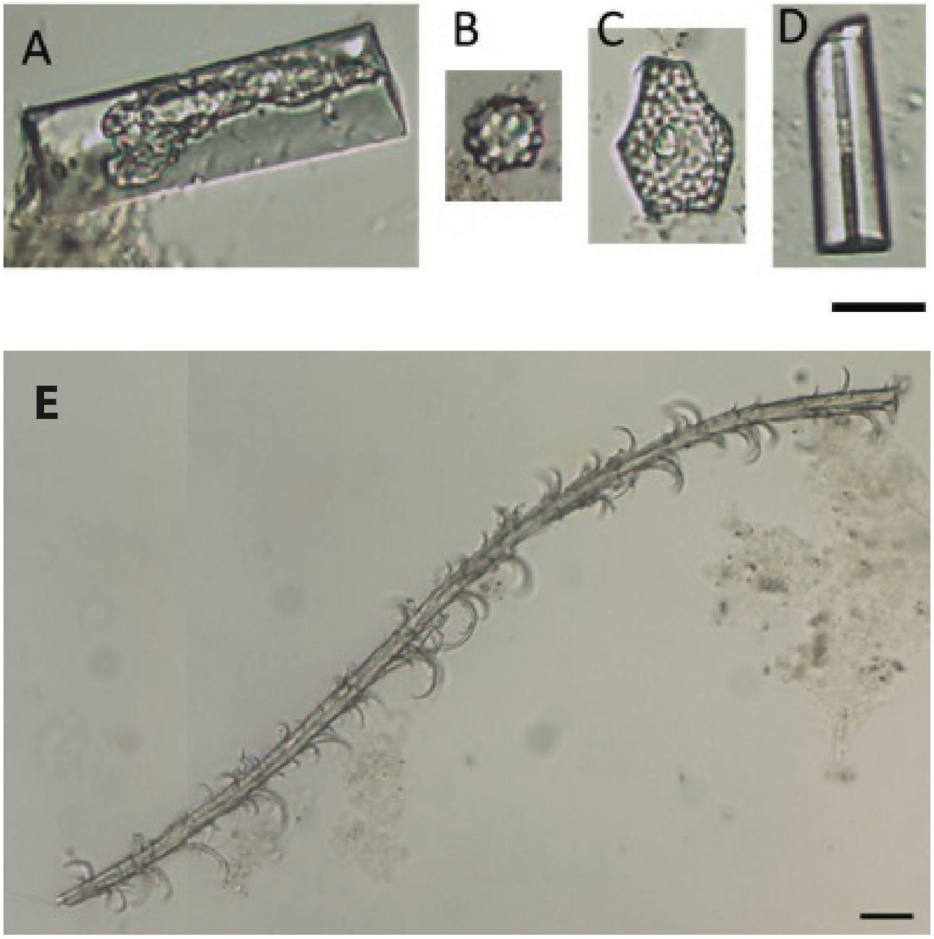

Supplementary Figure 22. Microfossils observed in dental calculus from Tongatapu. (**A-D**) Examples of phytoliths and sponge spicules recovered from Tongatapu. (**A**) Elongate psilate; (**B**) Spheroid echinate; (**C**) Polygonal scorbulate; (**D**) sponge spicule; scale bar is 20 µm. (**E**) Potential feather barbule from TON001.C; scale bar is 20 microns.
