# Supplementary material for: Exploring the potential of dental calculus to shed light on past human migrations in Oceania
Source: Nat Commun. 2024 Nov 24;15:10191. doi: 10.1038/s41467-024-53920-z (PMC11586442; doi:10.1038/s41467-024-53920-z)
Supplement: Supplementary file 3 — Description of Additional Supplementary Files [file 41467_2024_53920_MOESM3_ESM.pdf]

Description of Additional Supplementary Files

Supplementary Data 1. Overview of new data in this study and related laboratory and sequencing information

Supplementary Data 2. Overview of published data analyzed in this study

Supplementary Data 3. Pacific islands and ISEA dental calculus PCA species loadings

Supplementary Data 4. Mapping statistics for Tannerella forsythia

Supplementary Data 5. Mapping statistics for Anaerolineaceae bacterium oral taxon 439

Supplementary Data 6. Gubbins per-branch statistics for Tannerella forsythia

Supplementary Data 7. Gubbins per-branch statistics for Anaerolineaceae bacterium oral taxon 439

Supplementary Data 8. Reference genomes used for investigating authenticity of potential dietary finds

Supplementary Data 9. Microfossil findings
