# Supplementary material for: Exploring the potential of dental calculus to shed light on past human migrations in Oceania
Source: Nat Commun. 2024 Nov 24;15:10191. doi: 10.1038/s41467-024-53920-z (PMC11586442; doi:10.1038/s41467-024-53920-z)
Supplement: Supplementary file 5 — Reporting Summary [file 41467_2024_53920_MOESM5_ESM.pdf]

nature portfolio

Corresponding author(s): Christina Warinner
Last updated by author(s): Mar 25, 2024

Reporting Summary

Nature Portfolio wishes to improve the reproducibility of the work that we publish. This form provides structure for consistency and transparency in reporting. For further information on Nature Portfolio policies, see our Editorial Policies and the Editorial Policy Checklist.

Statistics

For all statistical analyses, confirm that the following items are present in the figure legend, table legend, main text, or Methods section.

n/a	Confirmed	
☐	☒	The exact sample size (*n*) for each experimental group/condition, given as a discrete number and unit of measurement
☐	☒	A statement on whether measurements were taken from distinct samples or whether the same sample was measured repeatedly
☐	☒	The statistical test(s) used AND whether they are one- or two-sided<br>*Only common tests should be described solely by name; describe more complex techniques in the Methods section.*
☐	☒	A description of all covariates tested
☐	☒	A description of any assumptions or corrections, such as tests of normality and adjustment for multiple comparisons
☐	☒	A full description of the statistical parameters including central tendency (e.g. means) or other basic estimates (e.g. regression coefficient) AND variation (e.g. standard deviation) or associated estimates of uncertainty (e.g. confidence intervals)
☒	☐	For null hypothesis testing, the test statistic (e.g. F, t, r) with confidence intervals, effect sizes, degrees of freedom and P value noted<br>*Give P values as exact values whenever suitable.*
☒	☐	For Bayesian analysis, information on the choice of priors and Markov chain Monte Carlo settings
☒	☐	For hierarchical and complex designs, identification of the appropriate level for tests and full reporting of outcomes
☒	☐	Estimates of effect sizes (e.g. Cohen's d, Pearson's r), indicating how they were calculated

Our web collection on statistics for biologists contains articles on many of the points above.

Software and code

Policy information about availability of computer code

Data collection	The scripts used for analysis and figure generation are available on github at https://github.com/ivelsko/pacific_calculus.
Data analysis	The scripts used for analysis and figure generation are available on github at https://github.com/ivelsko/pacific_calculus.

For manuscripts utilizing custom algorithms or software that are central to the research but not yet described in published literature, software must be made available to editors and reviewers. We strongly encourage code deposition in a community repository (e.g. GitHub). See the Nature Portfolio guidelines for submitting code & software for further information.

Data

Policy information about availability of data

All manuscripts must include a data availability statement. This statement should provide the following information, where applicable:
- Accession codes, unique identifiers, or web links for publicly available datasets
- A description of any restrictions on data availability
- For clinical datasets or third party data, please ensure that the statement adheres to our policy

The sequencing data for this study is deposited in the European Nucleotide Archive under accession PRJEB61887

Research involving human participants, their data, or biological material

Policy information about studies with human participants or human data. See also policy information about sex, gender (identity/presentation), and sexual orientation and race, ethnicity and racism.

Reporting on sex and gender	n/a
Reporting on race, ethnicity, or other socially relevant groupings	n/a
Population characteristics	n/a
Recruitment	n/a
Ethics oversight	n/a

Note that full information on the approval of the study protocol must also be provided in the manuscript.

Field-specific reporting

Please select the one below that is the best fit for your research. If you are not sure, read the appropriate sections before making your selection.

☐ Life sciences ☐ Behavioural & social sciences ☒ Ecological, evolutionary & environmental sciences

For a reference copy of the document with all sections, see nature.com/documents/nr-reporting-summary-flat.pdf

Ecological, evolutionary & environmental sciences study design

All studies must disclose on these points even when the disclosure is negative.

Study description	Metagenomic data for dental calculus samples from 102 ancient human individuals were investigated to reconstruct the microbial communities of ancient oral microbiomes from 11 Pacific islands. Patterns of microbial diversity and phylogeny were investigated and compared to worldwide oral microbiome data.
Research sample	Dental calculus from archaeological humans excavated from Pacific islands spanning the past 3,000 years
Sampling strategy	For previously excavated archaeological individuals with prior approval for genetic analysis from 11 Pacific islands, all individuals with calculus deposits were selected for inclusion in this study.
Data collection	Genetic data was generated at the MPI-EVA, the University of Oklahoma, and the University of Otago (details provided in SI). Data collection methods are described in detail in the SI.
Timing and spatial scale	Archaeological remains spanning the past 3,000 years
Data exclusions	None
Reproducibility	Adequate sample sizes were investigated at each site to achieve data reproducibility.
Randomization	Not relevant
Blinding	Not relevant; the study investigates microbiome variation across time and space

Did the study involve field work? ☐ Yes ☒ No

Reporting for specific materials, systems and methods

We require information from authors about some types of materials, experimental systems and methods used in many studies. Here, indicate whether each material, system or method listed is relevant to your study. If you are not sure if a list item applies to your research, read the appropriate section before selecting a response.

Materials & experimental systems

n/a	Involved in the study
☒	☐ Antibodies
☒	☐ Eukaryotic cell lines
☐	☒ Palaeontology and archaeology
☒	☐ Animals and other organisms
☒	☐ Clinical data
☒	☐ Dual use research of concern
☒	☐ Plants

Methods

n/a	Involved in the study
☒	☐ ChIP-seq
☒	☐ Flow cytometry
☒	☐ MRI-based neuroimaging

Palaeontology and Archaeology

Specimen provenance

Efate, Vanuatu. The Vanuatu Cultural Centre (VCC) and the Vanuatu National Cultural Council provided ethical oversight of the study of human remains from Efate. In addition, the leaseholder M.R. Monvoisin and family and the traditional landowners and population of Eratap Village provided support for the study of remains on their ancestral lands. Monica Tromp entered into a research agreement in 2013 with the Vanuatu Cultural Centre to conduct research on human dental calculus from multiple archaeological sites in Vanuatu. The permit number is 10071.

Flores, Lesser Sunda Islands, Indonesia. The Pain Haka cemetery on the island of Flores in Indonesia was discovered and excavated by archaeologist Jean-Christophe Galipaud and anthropologists Charles Illouz and Philippe Grangé in 2010 (Institute for Research for Development [IRD] and University of La Rochelle) under a research permit from the Province of Nusa Tengarra Timur. Further excavation and sampling in 2012 were made possible by a joint collaboration between IRD, Puslit Arkenas and University of Otago. Export permits were obtained from Puslit Arkenas and RISTEK (Foreign Research Permit Division, Ministry of Research and Technology/National Research and Innovation Agency). Local communities participated at all stages of the research and gave permission for the removal of samples for analysis. All samples were collected and prepared by Rebecca Kinaston and exported to Otago University for management.

Futuna, Vanuatu. The Vanuatu Cultural Centre (VCC) and the Vanuatu National Cultural Council provided ethical oversight of the study of human remains from Futuna. Monica Tromp entered into a research agreement in 2013 with the Vanuatu Cultural Centre to conduct research on human dental calculus from multiple archaeological sites in Vanuatu. The permit number is 10071.

Raiatea, Society Islands. Excavations at Taputapuatea on the island of Raiatea were undertaken in 1994 and 1995 by archaeologists of the Centre Polynésien des Sciences Humaines (Tahiti, Polynésie française), and the export permit for subsequent analysis was issued by Service de la Culture et du Patrimoine (Tahiti, Polynésie française) in 2014. More recent communication (March 2020) with Director of Direction de la Culture et du Patrimoine (Tahiti, Polynésie française), granted permission for human dental calculus analyses.

Rapa Nui, Chile. Archaeological remains were sampled in 2002 as part of John Dudgeon's (2008) dissertation research from collections excavated during the National Geographic Easter Island Anthropological Expedition. Led by George Gill of the University of Wyoming, Sergio Rapu, former curator of the Sebastian Englert Museum, and Claudio Cristino of the University of Chile, the expedition collected the skeletal material in several field seasons, ending in 1981. The skeletal remains were curated at the Museo Antropológico Padre Sebastián Englert (MAPSE), Rapa Nui. Approval for the collection of skeletal remains for analysis was approved by the Consejo de Monumentos Nacionales de Chile, and the Museo Antropológico Padre Sebastián Englert, under then-director Francisco Torres Hochstetter. Skeletal materials from which dental calculus was extracted were repatriated to Rapa Nui in 2009.

Taumako, Duff Islands, Solomon Islands. The skeletal remains from Taumako in the Solomon Islands were excavated in the early 1970s by Foss Leach and Janet Davidson. We do not have any record of the original research permit. The skeletal remains were curated at the Anatomy Dept in Otago University firstly by Professor Phil Houghton and then by Hallie Buckley. The skeletal remains were repatriated in 2009 to the Solomon Islands National Museum. At that time permission was given to retain bone and tooth samples for further destructive analyses by the Director Lawrence Kiko.

Tongatapu, Tonga. Excavations at Talasiu (Tongatapu) were directed by archaeologist Geoffrey Clark and human and cultural remains export were permitted by Tongan Traditions Committee (Komoti Talafakafonua), Nuku'alofa, Kingdom of Tonga (2016-2025). The excavations of human skeletal remains at Talasiu were conducted in consultation and with the agreement of the Lapaha community.

Uripiv, Vanuatu. The Vanuatu Cultural Centre (VCC) and the Vanuatu National Cultural Council provided ethical oversight of the study of human remains from Uripiv. Monica Tromp entered into a research agreement in 2013 with the Vanuatu Cultural Centre to conduct research on human dental calculus from multiple archaeological sites in Vanuatu. The permit number is 10071.

Vao, Vanuatu. The Vanuatu Cultural Centre (VCC) and the Vanuatu National Cultural Council provided ethical oversight of the study of human remains from Vao. Monica Tromp entered into a research agreement in 2013 with the Vanuatu Cultural Centre to conduct research on human dental calculus from multiple archaeological sites in Vanuatu. The permit number is 10071.

Viti Levu, Fiji. The skeletal remains from the Sigatoka Dunes site on the island of Viti Levu in Fiji were sampled in 2015 from prior excavations curated at the Fiji Museum, Suva, Fiji. The original excavations were conducted under the Sigatoka Salvage Archaeological Project by Simon Best in 1987 and 1988. Sampling of skeletal remains was approved by the Fiji Ministry of Education and the Immigration Department and by the Fiji Museum, and sampling was assisted by museum staff Sepeti Matararaba, Jone Balenaivalu, Elia Nakoro, Sakiusa Kataiwi, and Jotami Naqeletia. Funding for this research was provided by the Nation Science

Foundation of the United States, Award # SBS 1216310.

Watom, Bismarck Archipelago, Papua New Guinea. Excavations at Watom in 2008 and 2009 were directed by archaeologist Dr. Dimitri Anson. Research permits for the excavation included permissions for export of human and cultural remains for analysis. In 2008, Hallie Buckley obtained permission from Herman Mandui, Chief Archaeologist of the National Museum and Art Gallery of Papua New Guinea in 2008. We have also had more recent communications (2023) with the Director of the Museum, Alous Kuaso, granting permission for further DNA and other destructive analyses. The excavations of human skeletal remains on Watom Island were conducted in consultation and with the agreement of the Village community.

Specimen deposition	Minimal sampling was performed and analytical specimens collected for analysis were fully consumed during analysis.
Dating methods	No new dates are reported

☐ Tick this box to confirm that the raw and calibrated dates are available in the paper or in Supplementary Information.

Ethics oversight	Detailed information for each island regarding ethical oversight and permissions for analysis are provided in the section "Ethics approvals and research permissions" in the Methods section of the article.

Note that full information on the approval of the study protocol must also be provided in the manuscript.

Plants

Seed stocks	n/a
Novel plant genotypes	n/a
Authentication	n/a
